# *HuangqiGuizhiWuwu* Decoction Prevents Vascular Dysfunction in Diabetes via Inhibition of Endothelial Arginase 1

**DOI:** 10.3389/fphys.2020.00201

**Published:** 2020-03-25

**Authors:** Hong Cheng, Tian Lu, Jingya Wang, Yucen Xia, Xiaoshu Chai, Minyi Zhang, Yutong Yao, Na Zhou, Sisi Zhou, Xinyi Chen, Weiwei Su, Cunzhi Liu, Wei Yi, Yongjun Chen, Lin Yao

**Affiliations:** ^1^School of Pharmaceutical Sciences, South China Research Center for Acupuncture and Moxibustion, Guangzhou University of Chinese Medicine, Guangzhou, China; ^2^Department of Oncology, Guangdong Provincial Hospital of Traditional Chinese Medicine, Guangzhou University of Chinese Medicine, Guangzhou, China; ^3^Guangdong Key Laboratory of Plant Resources, School of Life Sciences, Sun Yat-sen University, Guangzhou, China; ^4^Acupuncture Research Center, Beijing University of Chinese Medicine, Beijing, China

**Keywords:** *HuangqiGuizhiWuwu* decoction, arginase 1, nitric oxide, diabetic vascular dysfunction, endothelial-dependent vasorelaxation

## Abstract

Hyperglycemia induces vascular endothelial dysfunction, which contributes to the development of vascular complication of diabetes. A classic prescription of traditional medicine, *HuangqiGuizhiWuwu* Decoction (HGWWD) has been used for the treatment of various cardiovascular and cerebrovascular diseases, which all are related with vascular pathology. The present study investigated the effect of HGWWD treatment in streptozocin (STZ)-induced vascular dysfunction in mouse models. *In vivo* studies were performed using wild type mice as well as arginase 1 knockout specific in endothelial cells (EC-A1^–/–^) of control mice, diabetes mice and diabetes mice treated with HGWWD (60 g crude drugs/kg/d) for 2 weeks. For *in vitro* studies, aortic tissues were treated with mice serum containing HGWWD with or without adenoviral arginase 1 (Ad-A1) transduction in high glucose (HG) medium. We found that HGWWD treatment restored STZ-induced impaired mean velocity and pulsatility index of mouse left femoral arteries, aortic pulse wave velocity and vascular endothelial relaxation accompanied by elevated NO production in the aorta and plasma, as well as reduced endothelial arginase activity and aortic arginase 1 expression. The protective effect of HGWWD is reversed by an inhibitor of nitric oxide synthesis. Meanwhile, the preventive effect of serum containing HGWWD in endothelial vascular dysfunction is completely blocked by Ad-A1 transduction in HG incubated aortas. HGWWD treatment further improved endothelial vascular dysfunction in STZ induced EC-A1^–/–^ mice. This study demonstrates that HGWWD improved STZ-induced vascular dysfunction through arginase 1 – NO signaling, specifically targeting endothelial arginase 1.

## Introduction

The number of diabetes patients is expected to increase from 415 million in 2015 to 640 million by 2040 globally (Gao et al., 2016). Vascular complication of diabetes is one of the most serious manifestations of the disease (Rask-Madsen and King, 2013), and the leading cause for this condition is vascular dysfunction including impaired vascular endothelial vasodilation, reduced vascular compliance, and slowed blood flow (Shi and Vanhoutte, 2017). The most well-established clinical advances in the prevention of vascular complication include the control of glucose, cholesterol and blood pressure, which can slow the progression of diabetic microvascular pathology and reduce the risk of cardiovascular disease (Ong et al., 2008). However, the effect of the above-mentioned treatment on vascular dysfunction is not ideal (Grunberger, 2017; Ali et al., 2018). Therefore, there is an urgent need for the discovery and development of new drugs for the treatment of diabetic vascular complication.

Traditional Chinese medicine has long been involved in the treatment of cardiovascular diseases. *HuangqiGuizhiWuwu* Decoction (HGWWD), originated from “Synopsis of the Golden Chamber,” is one of the main prescriptions for treating vascular disease in ancient China. Recent clinical evidence-based studies have shown that HGWWD has a positive therapeutic effect on various cardiovascular and cerebrovascular diseases. For example, HGWWD can improve cerebral blood flow in stroke patients and alleviate the subjective symptoms of diabetic peripheral neuropathy (Baiqing, 2015; Pang et al., 2016). Similarly, a recent study reported that HGWWD effectively treated lower extremity macroangiopathy in diabetic patients (Hu et al., 2018). However, the underlying mechanism behind the HGWWD prevention of diabetes-induced vascular complication is poorly understood.

Studies from diabetic mice models and human patients with cardiovascular disease have reported abnormal arginase activation in the vascular endothelium (Romero et al., 2008, 2012; Beleznai et al., 2011; Shemyakin et al., 2012). Arginase, a urea cycle enzyme, can reciprocally regulate nitric oxide (NO) production by nitric oxide synthase (NOS) through competition for their common substrate, L-arginine (Bhatta et al., 2017). There are two isoforms of arginases: arginase 1, located in the cytoplasm and arginase 2, largely present in the mitochondria. We and other previous research found that elevated vascular arginase activity, especially arginase 1, can impair normal vascular endothelial function in various cardiovascular disease models, for example, diabetes (Elms et al., 2013), atherosclerosis (Rabelo et al., 2015), hypertension (Toque et al., 2013), aging (Santhanam et al., 2007), coronary artery disease (Shemyakin et al., 2012), and ischemia-reperfusion (Jung et al., 2010). We recently reported that knocking out endothelial arginase 1 completely prevented obesity-induced vasculopathy, including endothelial-dependent dysfunction, arterial stiffening and vascular inflammation (Bhatta et al., 2017; Yao et al., 2017). All of these findings indicate that arginase 1, especially in endothelial cells, is a valuable target for the treatment of vascular disease.

In this study, we evaluated the therapeutic effect of HGWWD on diabetes-induced vascular dysfunction and investigated whether the ameliorative effect of HGWWD on diabetic vascular endothelial dysfunction is associated with endothelial arginase 1-NO signaling.

## Materials and Methods

Arginase1 knockout specific in endothelial cells mice (EC-A1^–/–^) are obtained from Dr. W.R. Caldwell Laboratory (Augusta University, United States). As described previously (Bhatta et al., 2017), EC-A1^–/–^ mice were generated by mice expressed Cre-Cadherin 5 (C57BL/6J × 129S1/SvImJ, Stock No. 017968, Jackson Laboratory) crossed with mice carrying floxed arginase 1 alleles (A1^*loxp/loxp*^,C57BL/6J xB6.Cg-Thy1^*a*^,Stock No. 008817, Jackson Laboratory). Littermate A1^*loxp/loxp*^ mice were used as control mice. C57BL/6J Wild type male mice (WT) were obtained from Jinan Peng Yue Experimental Animal Breeding Co., Ltd. (Jinan, China). All mice were housed under a 12 h light/dark cycle with *ad libitum* access to water and food. All experimental procedures in this study were performed according to Institutional Animal Care and Use Committee of Guangzhou University of Chinese Medicine (GZUCM). Diabetic mice were used in a model of streptozotocin (STZ, 50 mg/kg)-induced diabetes (Yao et al., 2013). After 8 weeks of diabetes progression, the mice from the Chinese formula treatment groups received *HuangqiGuizhiWuwu* Decoction (HGWWD) by daily gavage at different doses (60 g/kg/d of crude drugs) for another 2 weeks with or without co-treatment with L-Name (NOS inhibitor) (Anea et al., 2012). Blood glucose and body weight levels of each animal were measured at the first day, second, eighth, and tenth weeks of STZ injection. Both hemodynamic function and vascular wall function were assessed by ultrasound as previously described (Bhatta et al., 2015; Kenwright et al., 2015; Faita et al., 2018; Esfandiarei et al., 2019). Vascular endothelial function was measured by myograph as previously described (Yao et al., 2013). Vascular NO production was determined using the fluorescent NO indicator4, 5-diaminofluorescein diacetate (DAF-2 DA) (Yao et al., 2013). Arginase activity in aortic lysates or plasma samples was determined as previously described (Yao et al., 2013). The mRNA levels of arginase 1 and arginase 2 were measured by quantitative reverse transcription PCR (Q-PCR) as previously described (Bhatta et al., 2017). Data are shown as mean ± SEM. The number of experiments is indicated by “n.” Statistical differences were determined using analysis of variance (ANOVA) followed by Tukey post-test. *P* < 0.05 were taken as significant. Detailed methods are provided in Supplementary Material and Methods.

## Results

### HGWWD Treatment Prevents Diabetes-Induced Impairment of the Vascular System

**FIGURE 1:**
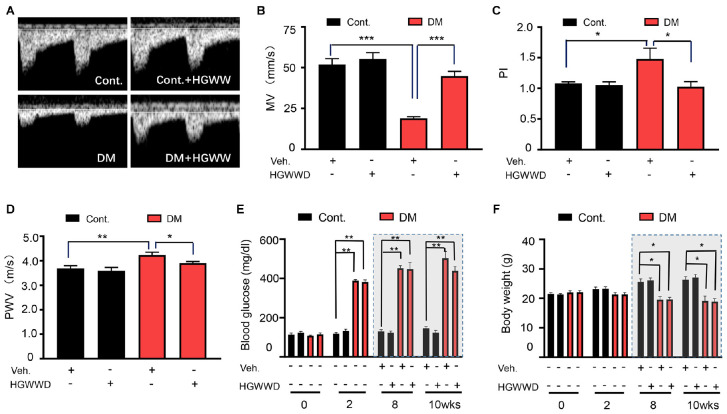
HGWWD prevented the impairment of vascular system without altering blood glucose and body weight in STZ mice. **(A)** Representative images of Pulsed Doppler spectral waveform of left femoral arteries in mice. **(B–D)** The analysis of MV and PI of left femoral arteries and aortic PWV in mice by ultrasound. **(E)** Blood glucose level and **(F)** body weight at 0, 2nd, 8th, and 10th weeks of STZ injection with or without HGWWD treatment. MV, mean velocity; PI, pulsatility index; PWV, pulse wave velocity; STZ, streptozotocin; Cont., non-diabetic normal mice; DM, diabetic mice induced by STZ; HGWWD, *HuangqiGuizhiWuwu* Decoction. Values are presented as mean ± SEM, *P < 0.05, **P < 0.01, and ***P < 0.001, *n* = 6–8 mice/group.

The function of the vascular system can be regulated by both hemodynamic function and vascular wall function (Pugsley and Tabrizchi, 2000; Lee, 2013). In diabetes patients and animal models, abnormal characteristics of hemodynamic and vascular wall function have been reported *in vivo* studies by non-invasive ultrasonography (Cardoso et al., 2015; Faita et al., 2018; Chirinos et al., 2019; Kozera et al., 2019). In this study, we chose mean velocity (MV) to represent the kinetic energy of the blood flow, pulsatility index (PI) to evaluate the vascular resistance and pulse wave velocity (PWV) to predict vascular wall stiffness (Panaritis et al., 2005; Lee, 2013; Alman et al., 2018), which are commonly used in evaluating the function of vascular system. The effect of HGWWD on the function of vascular system is indirectly evaluated by these three parameters after 2 weeks of HGWWD treatment in diabetic mice (Supplementary Figures S1, S2). As shown in Figures 1A–D, STZ reduced the values of MV and increased PI values of left femoral arteries in mice, compared with control mice. We also found that the values of aortic PWV were elevated in STZ mice (Figure 1D). Furthermore, HGWWD administration improved values of MV, PI, and PWV caused by STZ injection (Figures 1A–D). To determine whether the effect of HGWWD happens through regulation of metabolic syndrome, we tested the change in blood glucose and body weight at different points in time (1st day, 2nd day, 8th week, and 10th week) after STZ injection, with and without HGWWD. Mice started to show a large increase in glucose levels and a reduction in body weight at 2 weeks after STZ injection. Unexpectedly, HGWWD did not alter glucose levels or body weight in mice with STZ (Figures 1E,F). Taken together, these results suggest that the protection that HGWWD provides against the impairment of both hemodynamic function and vascular wall function is not secondary to the improvement of blood glucose and body weight.

### HGWWD Treatment Restores Diabetes-Induced Vascular Endothelial Dysfunction

**FIGURE 2:**
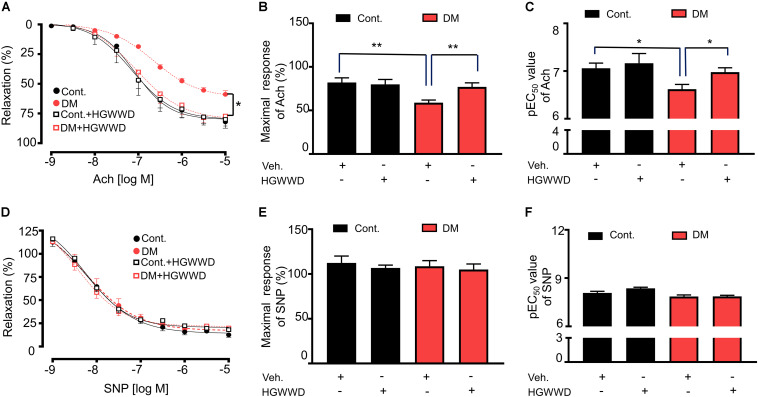
HGWWD prevented STZ-induced decreased in endothelium-dependent vasorelaxation to Ach. **(A)** Effects of HGWWD presented in relaxation curve and **(B)** the value of maximal relaxation (E_*max*_) and **(C)** pEC_50_ (the negative logarithm of EC_50_) of Ach. **(D)** Effect of HGWWD in endothelium-independent vasorelaxation curve and **(E)** the value of E_*max*_ and **(F)** pEC_50_ of sodium nitroprusside (SNP). Values are presented as mean ± SEM, *P < 0.05 and **P < 0.01, *n* = 10–16 samples from 5 to 8 mice/group.

The dysfunction of endothelium located in the flow-tissue interface is considered as the initiation of vascular disorder (Rahman et al., 2007), which contributes to atherosclerosis formation (Gimbrone and Garcia-Cardena, 2016), vascular inflammation (Yang et al., 2016) and vascular remodeling (Vandersmissen et al., 2015; Hao et al., 2019). We previously found that there is significant vascular impairment at 8 weeks in the STZ mouse model (Yao et al., 2013). To determine the effects of HGWWD on vascular endothelial dysfunction in diabetes, we analyzed the acetylcholine (Ach)-induced vasorelaxation response in aortas from control and STZ-induced mice with or without HGWWD. As shown in Figures 2A–C, STZ induction impaired vasorelaxation responded to Ach in aortas, presented by a decrease in the maximal relaxation (E_*max*_) value and an increase in EC_50_ in response to Ach from diabetic mice compared with controls. Intriguingly, HGWWD restored deficits of endothelial-dependent vasorelaxation in diabetic mice. To determine whether smooth muscle function is involved in the effect of HGWWD, we measured endothelial-independent relaxation to the NO donor sodium nitroprusside (SNP). As Figures 2D–F shows, curves of SNP were normal in all groups. All of these data indicate that HGWWD prevented diabetic vascular endothelial dysfunction by targeting the vascular endothelium.

### HGWWD Treatment Prevents Diabetes-Induced Vascular Endothelial Dysfunction Through NO Signaling

**FIGURE 3:**
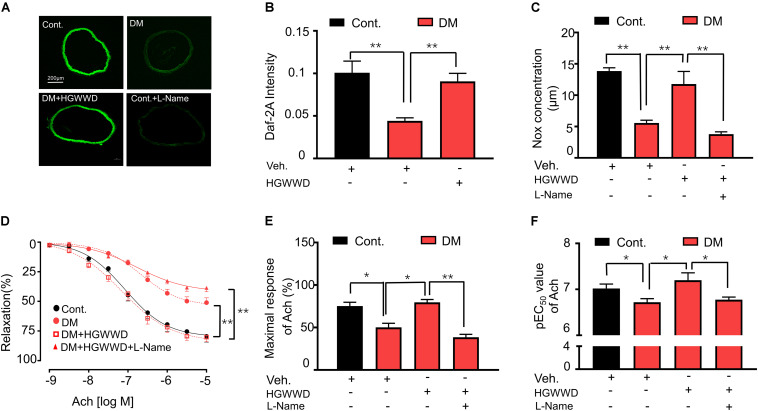
The prevention of HGWWD in vascular endothelial dysfunction was blocked by the inhibition of nitric oxide production. **(A)** Representative images of 4, 5-diaminofluorescein diacetate (DAF-2 DA) in aortic rings from all groups, corrected for fluorescence in the presence of L-Name. **(B)** Fluorometric analysis of DAF2-DA loaded aortic rings. **(C)** Total NO bioavailability (NOx) levels in plasma. **(D)** endothelium-dependent vasorelaxation curve, **(E)** the value of E_*max*_ and **(F)** pEC_50_ of Ach from all groups. Values are presented as mean ± SEM, *P < 0.05 and **P < 0.01, *n* = 8–16 samples from 4 to 8 mice/group.

The ability of the endothelium to synthesize NO is a key indicator for predicting and evaluating vascular function (Behrendt and Ganz, 2002). To investigate whether the protection of HGWWD in vascular endothelial dysfunction is related to vascular NO production, first we determined the NO levels in the aortas by DAF-2 fluorescence intensity (indicator of available NO). As shown in Figures 3A,B, the intensity of DAF-2 fluorescence was significantly reduced in diabetic mice compared with control mice, and the reduction in NO production was reversed by HGWWT. To determine whether the effect of HGWWT on vascular endothelial dysfunction requires NO signaling, diabetic mice were co-treated with HGWWD and L-Name (NOS inhibitor). We found that L-Name remarkably abolished the potentiating effect of HGWWD in plasma NO bioavailability in diabetic mice (Figure 3C). To further test whether altered plasma NO levels by HGWWD are related to the promotion of vascular endothelial function, we examined the vascular relaxation in response to Ach. L-Name treatment blocked the protective effect of HGWWD in diabetes-induced endothelial dysfunction, shown by reduced E_*max*_ value and elevated EC_50_ in response to Ach (Figures 3D–F). All of these results indicate that HGWWD treatment improved diabetes-induced vascular endothelial dysfunction through NO signaling.

### HGWWD Alleviates the Level of Endothelial Arginase Activation in Diabetic Aortas

**FIGURE 4:**
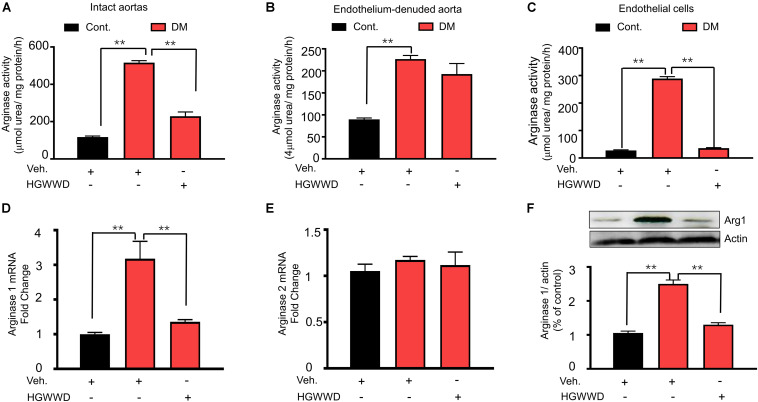
HGWWD attenuated endothelial arginase activity and aortic arginase1 expression in STZ mice. The values of arginase activity in **(A)** intact aortas, **(B)** endothelium-denuded aortas and **(C)** endothelial cells of aortic tissues. The mRNA levels of **(D)** arginase 1 and **(E)** arginase 2 in aorta rings. **(F)** The expression of arginase 1 in aortic rings. Values are presented as mean ± SEM, **P < 0.01, *n* = 8–12 samples from 4 to 6 mice/group.

To assess whether endothelial arginase activation is involved in the effects of HGWWD, arginase activity was analyzed in aortas and endothelial cells. The arginase activity in the endothelium was calculated by the subtraction of values for the endothelium denuded aortas from values of vascular tissues with an intact endothelial layer (Yao et al., 2013). As shown Figures 4A–C, arginase activity was largely enhanced in vascular endothelial cells of diabetic mice compared with non-diabetic, which was blocked by HGWWD. Consistently, HGWWD attenuated the increased arginase activity of the intact aortas as well. To identify which isoforms of arginase are important for the effect of HGWWD, we firstly determined the mRNA levels of arginase 1 and arginase 2 in aortas. HGWWD blunted the elevation of aortic arginase 1 mRNA levels induced by STZ (Figure 4D), but there was no difference in arginase 2 mRNA levels of all three groups (Figure 4E). Similarly, western blot analysis showed that STZ caused the elevation of arginase 1 protein level in the aorta, which can be reduced by HGWWD treatment (Figure 4F). Together, these findings indicate that HGWWD prevented STZ from increasing arginase activity and arginase 1expression, especially in endothelial cells.

### Arginase 1 Overexpression Reversed the Effect of HGWWD on HG-Induced Vascular Endothelial Dysfunction

**FIGURE 5:**
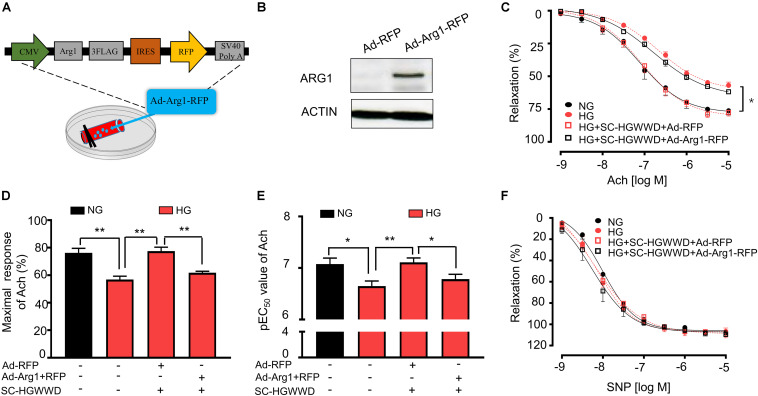
The prevention of HGWWD in vascular endothelial dysfunction was blocked by over expression of arginse 1 in high-glucose (HG) – incubated aortas. **(A)** Illustration of aortic transduction with Ad-Arg1-RFP; **(B)** The arginase 1 expression in aortic tissues after Ad-RFP and Ad-Arg1-RFP groups, Ad-RFP serves as control; **(C)** Endothelium-dependent vasorelaxation curve and **(D)** the value of E_*max*_ and **(E)** pEC_50_ of Ach in NG, HG and HG + SC-HGWWD incubated aortas with or without ad-Arg1-RFP. **(F)** endothelium-independent vasorelaxation curve of SNP. Ad-Arg1-RFP: aortic transduction with adenoviral vector carrying wild type arginase 1 with red fluorescence protein; Ad-RFP, aortic transduction with adenovirus transduction in RFP adenoviral vector; NG, normalglucose; HG, high glucose; SC-HGWWD, Serum containing HGWWD. Values are presented as mean ± SEM, *P < 0.05 and **P < 0.01, *n* = 10–12 samples from 5 to 6 mice/group.

We then determined whether reduced arginase 1 expression is the key mechanism for the HGWWD treatment by using adenoviral transduction to overexpress arginase 1 in isolated aortas (Figure 5A). As shown in Figure 5B, western blot analysis confirmed that arginase 1 expression was significantly increased in the aorta with arginase 1 transduction. To mimic hyperglycemia conditions, isolated aortas were exposed to Kreb’s buffer containing normal glucose (NG) and high glucose (HG) *ex vivo*. Aortas incubated with HG for 24 h started to exhibit significant vascular endothelial dysfunction (Supplementary Figure S3). Predictably, overexpression of arginase 1 blocked the effect of HGWWD on the impairment of vascular E_*max*_ and EC_50_ values (Figures 5C–E). In contrast, vasorelaxant responses to SNP were not different among all the groups (Figure 5F). These data further support the idea that HGWWD prevents vascular endothelial dysfunction through reducing vascular arginase 1.

### Arginase 1 in Endothelial Cells Is Critical for HGWWD on Vascular Endothelial Dysfunction

**FIGURE 6:**
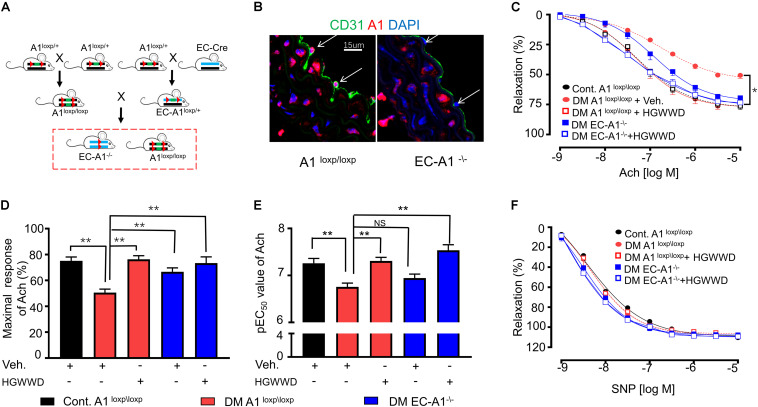
HGWWD prevented STZ-induced vascular endothelial dysfunction through endothelial specific arginase 1. **(A)** The generation of arginase 1 deletion in endothelial cells (EC-A1^–\–^) mice; **(B)** Immunostaining in aortic sections of (EC-A1^–\–^ and its littermates (A1^*loxp/loxp*^) mice of control by antibodies of endothelial marker (CD31) and arginase 1; **(C)** Endothelium-dependent relaxation curve; **(D)** the values of E_*max*_ and **(E)** pEC_50_ of Ach in mice of EC-A1^–\–^, A1^*loxp/loxp*^, diabetes and diabetes treated with or without HGWWD treatment; **(F)** endothelium-independent vasorelaxation curve to SNP. Values are presented as mean ± SEM, *P < 0.05 and **P < 0.01, *n* = 10–12 samples from 5 to 6 mice/group.

We next determined whether endothelial arginase 1is involved in the prevention of HGWWD on vascular endothelial dysfunction in STZ mice models. The mating strategy of endothelial-specific arginase 1 knockout (EC-A1^–/–^) mice is shown in Figure 6A. Immunostaining evidence indicates that arginase 1 immunoreactivity was abolished inEC-A1^–/–^ aortic sections (Figure 6B). In the STZ model, lack of arginase 1 in endothelial cells increased vascular E_*max*_ compared with aortas from littermate controls (A1^*loxp\loxp*^), but it did not alter the values of EC_50_ (Figures 6C–E). These results indicate that lost arginase 1 in endothelial cells attenuates STZ-caused vascular endothelial dysfunction. Interestingly, lacking endothelial arginase 1 cannot further increase the effect of HGWWD on improved vascular E_*max*_ or EC_50_ in STZ mice (Figures 6C–E). In contrast, the vasorelaxation curves of SNP were similar among all groups (Figure 6F). These data provide further support to the notion that HGWWD protects STZ-induced vascular endothelial dysfunction via endothelial arginase 1.

## Discussion

In this study, we found that (1) treatment with HGWWD for 2 weeks protected against diabetes-induced impairment of both hemodynamic function and vascular wall function, but did not alter blood glucose levels and body weight in diabetic mice; (2) HGWWD prevented diabetes-caused vascular endothelial dysfunction through arginase 1-NO signaling; (3) endothelial arginase 1 is a critical target for the prevention of HGWWD on vascular endothelial dysfunction in diabetic models.

HGWWD is used in the treatment of various cardiovascular and cerebrovascular diseases, including diabetic lower extremity macroangiopathy (Hu et al., 2018), diabetic peripheral neuropathy (Liu et al., 2019), and stroke (Li, 2015). Similarly, we found that HGWWD significantly ameliorated vascular wall and hemodynamic dysfunction, especially endothelial dysfunction in STZ-induced mice (Figures 1, 2), suggesting that HGWWD is effective in treating the disorder of vascular system caused by diabetes. Controlling blood glucose levels is one of the most common therapies for slowing the progress of vascular disease in diabetic patients (Zhang et al., 2019). However, our present study shows that HGWWD has no effect on blood glucose in diabetic mice (Figures 1D,E), which is supported by several clinical studies (Liu, 2005; Bian, 2010; Lu and You, 2010; Pang et al., 2016). One study reported that HGWWD can reduce the glucose levels of diabetic patients (Chen, 2019), but the reasons for this discrepancy are unclear. We speculate the different effects of HGWWD on blood glucose may be attributable to differences in dosage or duration of treatment. Nevertheless, this study provides evidence to support that this traditional Chinese formula can significantly attenuate vascular complications in diabetes patients.

In this study, we reported that HGWWD treatment significantly restored aortic vascular endothelial dysfunction through NO signaling (Figure 3). Abnormal NO levels have been reported in vascular pathological conditions (Bondonno et al., 2016), and intervention in NO signaling can effectively prevent various vascular disorders, including endothelial dysfunction, vascular inflammation and vascular stiffness (Bondonno et al., 2016; Batko et al., 2019; Chen et al., 2019). Furthermore, excessive arginase activation can down-regulate NO production by reducing NOS availability through competition for their common substrate L-arginine (Bhatta et al., 2017; Zhou et al., 2018). This notion is supported by our results showing that HGWWD decreased endothelial arginase activity and aortic arginase 1 expression in STZ mice (Figure 4). Moreover, overexpression of arginase 1 in the aorta can block the effect of HGWWD in diabetic mice (Figure 5) and the lack of endothelial arginase 1 cannot further improve the vascular endothelial function in the HGWWD-treated diabetic group (Figure 6). Thus, for the first time, we demonstrated that HGWWD prevented STZ-induced vascular dysfunction through arginase 1-NO signaling in vascular endothelial cells. Similarly, some other traditional medicines treated the vascular pathology involved in mechanisms with regulating vascular endothelium dysfunction and NO production, including Yangxin Decoction (Li et al., 2011), Taohong Siwu Decoction (Zhang, 2014), Shixiao San (Wang X. et al., 2015), Ling-Yang-Gou-Teng-decoction (Zhao et al., 2018). Moreover, our previous study also found that Xiao-Shen-Formula prevented the impairment of renal microvascular remodeling via inhibiting arginase activation (An et al., 2018). Altogether, our study further confirms that endothelial Arginase-NO signaling is a valuable therapeutic target in the invention of vascular disease.

There is wide belief that traditional Chinese multi-component formulas act on multiple targets. Besides arginase 1-NO signaling in endothelial cells, our results revealed that there should be other mechanisms involved the effect of HGWWD on the protection of vascular impairment in the STZ model. We found that HGWWD has better effect in preventing vascular endothelial function of diabetes than knocking out endothelial arginase 1 alone (Figure 6). Furthermore, there are other pharmacological effects of HGWWD, including the reduction of oxidative stress levels in diabetic neuropathy and myocardial ischemia-reperfusion injury in rats (Qi et al., 2013; Wang Y.-C. et al., 2015; Zhang et al., 2017), and anti-inflammation in nephropathy mice or arthritis in rats (Huang et al., 2009; Li et al., 2016; Liu, 2017; Liu et al., 2018). It is conceivable that the findings of this paper could shed light on the pharmacological mechanisms of HGWWD treatment, particularly in targeting endothelial arginase 1.

## Data Availability Statement

The datasets generated for this study are available on request to the corresponding author.

## Ethics Statement

The animal study was reviewed and approved by the Institutional Animal Care and Use Committee of Guangzhou University of Chinese Medicine (GZUCM).

## Author Contributions

LY, YC, WS, CL, and WY conceived and designed the protocol. HC, TL, JW, YX, XCha, MZ, YY, NZ, SZ, and XChe performed the experiments. LY, YC, HC, and TL analyzed the data. LY and YC wrote the manuscript. All the authors reviewed and approved the submitted version of the manuscript.

## Conflict of Interest

The authors declare that the research was conducted in the absence of any commercial or financial relationships that could be construed as a potential conflict of interest.

## Supplementary Material

FIGURE S1 The experimental schedule of STZ model with HGWWD treatment. STZ, streptozotocin; HGWWD, *HuangqiGuizhiWuwu* Decoction; NO, nitric oxide.

FIGURE S2 Schematic diagram of the measurement of mouse left femoral artery and aortic by ultrasound system. **(A)** Schematic diagram of mouse left femoral artery under the digital ultrasound platform. **(B)** The schematic diagram of mouse left femoral artery under the PW-mode combining with color flow Doppler imaging. **(C)** Schematic diagram of the analysis of peak systolic velocity (PSV)and end diastolic velocity (EDV) of mouse left femoral artery. **(D)** Schematic diagram of the analysis of mean velocity (MV) of mouse left femoral artery. **(E)** The schematic diagram of the measurement of pulse wave velocity (PWV). **(F)** The image of aorta under the M-mode. **(G)** Schematic diagram of the analysis of PWV under the enlarged image aortic vertical section from the part labeled by yellow dotted line.

FIGURE S3 **(A–C)** Aortic endothelium-dependent relaxation curve, **(D)** maximal relaxation and **(E)** pEC_50_ of Ach in HG incubation for 12, 24, and 36 h. Values are presented as mean ± SEM, ^∗^*P* < 0.05 and ^∗∗^*P* < 0.001, *n* = 10 samples from 5 mice/group.
