# Erratum: *HuangqiGuizhiWuwu* Decoction Prevents Vascular Dysfunction in Diabetes via Inhibition of Endothelial Arginase 1

**DOI:** 10.3389/fphys.2021.650179

**Published:** 2021-01-20

**Authors:** Frontiers Production Office

**Affiliations:** Frontiers Media SA, Lausanne, Switzerland

**Keywords:** *HuangqiGuizhiWuwu* decoction, arginase 1, nitric oxide, diabetic vascular dysfunction, endothelial-dependent vasorelaxation

Due to a production error, there was a mistake in Figure 1 as published. The wrong figure appeared. The corrected Figure 1 appears below. The publisher apologizes for this mistake.

**Figure 1:**
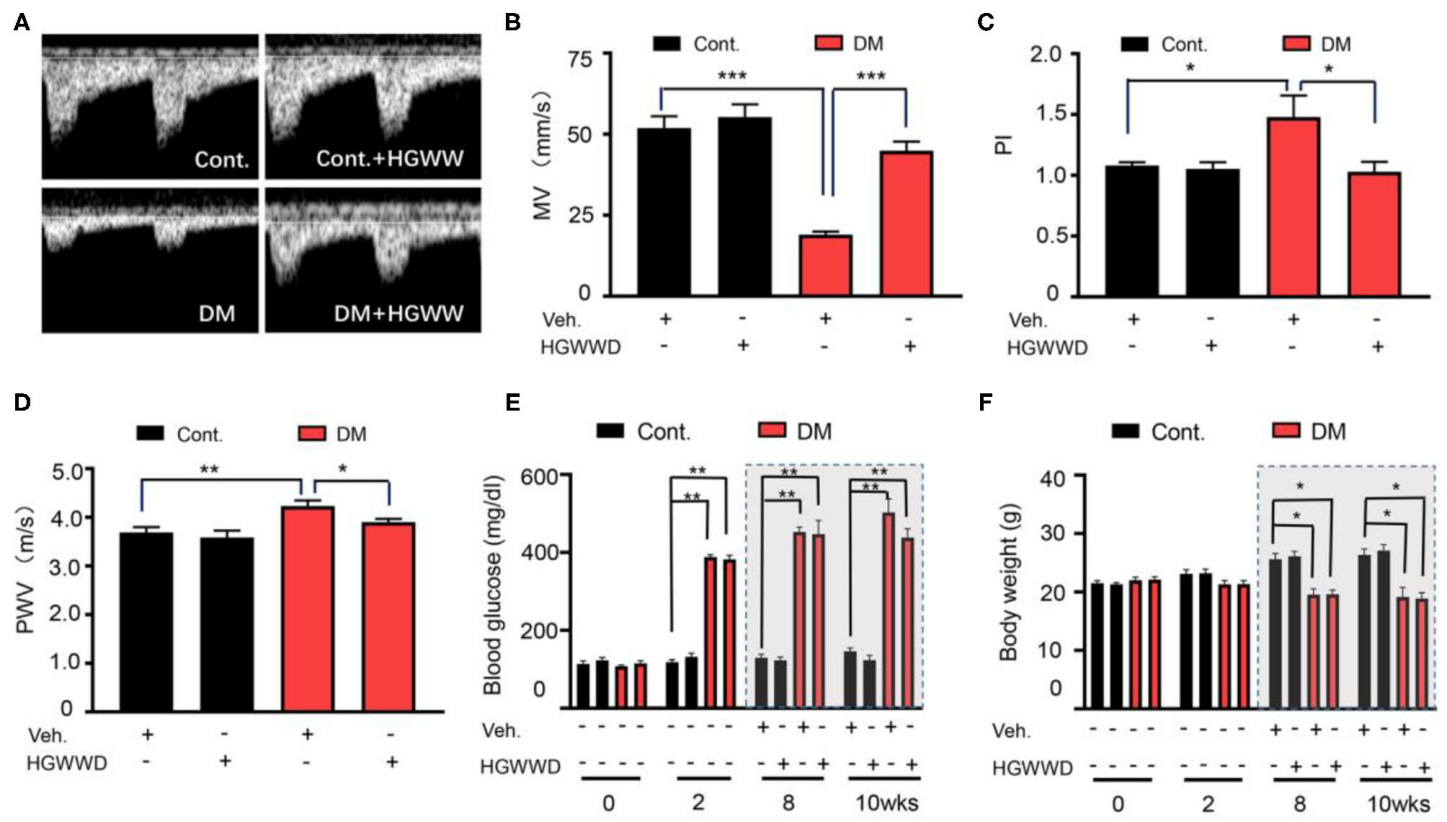
HGWWD prevented the impairment of vascular system without altering blood glucose and body weight in STZ mice. **(A)** Representative images of Pulsed Doppler spectral waveform of left femoral arteries in mice. **(B–D)** The analysis of MV and PI of left femoral arteries and aortic PWV in mice by ultrasound. **(E)** Blood glucose level and **(F)** body weight at 0, 2nd, 8th, and 10th weeks of STZ injection with or without HGWWD treatment. MV, mean velocity; PI, pulsatility index; PWV, pulse wave velocity; STZ, streptozotocin; Cont., non-diabetic normal mice; DM, diabetic mice induced by STZ; HGWWD, *HuangqiGuizhiWuwu* Decoction. Values are presented as mean ± SEM, ^*^P < 0.05, ^**^P < 0.01, and ^***^P < 0.001, *n* = 6–8 mice/group.

The original article has been updated.

