# Supplementary material for: HuangqiGuizhiWuwu Decoction Prevents Vascular Dysfunction in Diabetes via Inhibition of Endothelial Arginase 1
Source: Front Physiol. 2020 Mar 25;11:201. doi: 10.3389/fphys.2020.00201 (PMC7109290; doi:10.3389/fphys.2020.00201)
Supplement: Supplementary file 1 [file Data_Sheet_1.pdf]

Supplementary Materials and Methods

Animal Studies

All diabetic mice were injected by STZ (50mg/kg, No: V900890, Sigma, Saint Louis, USA) every other day for up to three injections. Non-diabetic mice received citrate buffer (the vehicle of STZ, pH = 4.5) in the same manner as in the diabetic mice. Mice with blood glucose levels > 350 mg/dL were considered as diabetes. After 8 weeks of diabetes progression, the mice with Chinese formula treatment groups received *HuangqiGuizhiWuwu* Decoction (HGWWD) by daily gavage at dose 60 g/kg/d of crude drugs for another 2 weeks with or without co-treating L-Name (NOS inhibitor, 1mg /ml in drinking water, No: N5751, Sigma, Saint Louis, USA). Blood glucose and body weight levels of each animal were measured at the first day, second, eighth and tenth weeks of STZ injection. The dosage of HGWWD (60 g/kg/d of crude drugs) in this study was calculated based on the dosage of human (4.5g/kg/d of crude drugs) in clinical practice.

Preparation for the Lyophilized Powder of HGWWD

According to the records in the Essentials from the Golden Cabinet, which is one of the classical ancient Chinese medicine books, the medicinal materials were weighed according to the ratio of *Astragalusmembranaceus (huangqi*): *cinnamon twig* (*guizhi*): *Radix Paeoniae Alba* (*baishao*): *ginger* (*shengjiang*): *jujube* (*dazao*)= 1:1:2:1:1. All herbal medicines were purchased from Kangmei Pharmaceutical Co. Ltd., China. These herbs were extracted twice by refluxing with 10 times of water (volume/weight) for 1h each time and fragrances were collected for 3 times during the extraction. Then, the extracted solution was filtered twice. The filtered extracts were mixed together and then concentrated to the relative density for 1.08 g/ml. The concentrated liquid mixed with the fragrant evenly was pre-frozen at -80°C and freeze-dried for 24 h. The plate-like extracts were crushed into powder and stored at -80°C for reserving. Before using, the lyophilized powder of HGWWD was dissolved by purified water according to the dosage.

Preparation of Serum-containing HGWWD

Male mice (C57BL/6J) took HGWWD (60 g crude drugs /kg/d) or purified water by daily gavage for 2 weeks and the blood was collected from sacrificed mice 2 h after final dose. The blood samples were centrifuged at 2500 rpm for 15min at 4°C, collected serum was incubated in a 56°C water for 30min for inactivation and stored at -80°C before using (An et al. 2018).

Aortic Tissue Preparation

After eight-weeks of diabetes, animals were anesthetized with ketamine HCl (100 mg/kg) and xylazine (10mg/kg) (i.p.). When adequacy of anesthesia was reached as indicated by the disappearance of pedal withdrawal reflex, the isolated aorta from sacrificed mice was placed in chilled Krebs buffer (NaCl, 118 mM; NaHCO3, 25 mM; glucose, 5.6 mM; KCl, 4.7 mM; KH2PO4, 1.2 mM; MgSO4·7H2O, 1.17 mM and

$CaCl2 \cdot 2H2O$, 2.5 mM). Residual blood and perivascular fat was removed before experiments. Half of each aorta was denuded of endothelial layer by gently rubbing the intimal surface with a needle, which is used for the calculation of endothelial arginase activity. The absence of endothelium in aorta was determined by no responseto acetylcholine (ACh, 10^{-6}M) during the function study. The other half intact aorta was also left for the arginase activity and arginase 1 expression measurement.

Vivo ultrasound imaging and assessment

Study of vascular wall and hemodynamic funtion in both left femoral artery and aorta was performed using a Vevo 2100 Ultrasound Imagine system (Fujifilm Visualsonics ,Canada), which has an ultrasonic coverage of 22-55MHz and a central frequency of 40MHz. Measurements were taken in random order from mice with numbers labeled by only one operator (Esfandiarei et al. 2019; Faita et al. 2018; Kenwright et al. 2015). Briefly, Mice were anesthetized in an induction chamber containing 4% isoflurane and 1 L/min of medical oxygenfor 1–1.5 mins, then concentration of isofluranewas adjusted to 1-2% to keep the miceanesthetized during the measurements. Mice hair of left legs was removed with depilatory cream in the supine position. During the experiment, heart rate, electrocardiogram (ECG), and respiratory rate of mice were measured by the four ECG electrodes embedded in the operating platform. The body temperature was maintained at 36–38°C and monitored by a rectal probe throughout the protocol and the heart rate was maintained at 400-450bpm. The ultrasound probe (MS550D) was placed at the main branch of left femoral arteryfrom the abdominal aortaand parallelled to the longitudinal section of the blood vessel after the heating platform in the head side of contralateral femoral artery was pressed down to expose the objective vessel (revised Supplement Fig. 2A). Firstly, the specific location and contour of the blood vessels were detected under B-mode and the direction of blood flow was determined in color doppler mode through adjusting the angle and direction of the probe. Then Pulsed Wave Doppler (PW-mode) images were acquired immediately and the data was recorded automatically by the on-board software (revised Supplement Fig. 2B). Doppler gain was adjusted until a few pixels were at maximum whiteness in order to reduce the background value of the image. In the PW-mode file, the Vevo LAB software tool was used to markthe left femoral artery blood flow waveform to obtain the left femoral artery mean velocity (MV), the left femoral artery systolic peak velosity (PSV) and left femoral artery end-diastolic velocity (EDV) (revised Supplement Fig. 2C and D). Left femoral pulsatility index (PI) was calculated using the formula:(PSV-EDV)/ MV. For each value, three cycles were measured and averaged.

Aortic pulsewaves velocity (PWV) of each mousewasdeterminedat the same time (Bhatta et al. 2015). Briefly, mice abdomimal hair was removed in the supine position. The specific location and contour of the aorta vessels were detected under B-mode and the direction of blood flow was determined in color doppler mode. Aortic pulse waves were assessed at sits of aorta: the aortic arch and abdominal aorta proximal to iliac bifurcatio. The distance between two sits was measured by the scale after aorta was isolated. The arrival times of a flow wave at two locations along the aorta

between two sits werecalculated by referencing the points at the R-wave of the ECG under the M-mode. PWV (m/s) was calculated by dividing the distance (L) by the difference between two arrival times (T1-T2) with an average of 3-5 cardiac cycles (revised Supplement Fig. 2E-G). For all the measurements, the total scanning time for each mouse was limitied to less than 30 mins,includingoptimization of the probe position and alignment, adjustment of machine settings and trial recordings.

Vascular Function

Vascular function was measured by myograph (No: DMT620, Danish Myo Technology, Aarhus, Denmark) as previously described(Yao et al. 2013) and isometric force was recorded using a PowerLab/8SP data acquisition system (Software Chart, Version 5, AD Instrument, Colorado Springs, CO, USA). Aorta tissue was placed in cold Krebs buffer (pH 7.4) and cut into 2mm rings. Rings were mounted in 5ml baths of myograph for 1 h equilibration, which were filled with Krebs solution (37°C) under resting tension of 5.0mN and bubbled with 95% O2 and 5% CO2. Isometric force was recorded using a Power Lab system (AD Instruments, Colorado Springs, CO, USA). The ability of the preparation to cause contraction was assessed by applying 80mM KCl solution. Thereafter, cumulative concentration-response curves to acetylcholine (Ach: 10^{-9} to 10^{-5}M, endothelium-dependent vasodilator, No: A2661, Sigma, Saint Louis, USA) or NO donor, sodium nitroprusside (SNP: 10^{-9} to 10^{-5}M, endothelium-independent vasodilator, No:71778, Sigma, Saint Louis, USA), were obtained in rings precontracted with phenylephrine (PE: 10^{-6}M, No:P1250000, Sigma, Saint Louis, USA).

Tissue Culture Studies

Aortic tissues were isolated from male wild type mice which are 10 weeks old. Fresh tissues were cultured in complete medium containing M199 (GibcoThermo Fisher, Waltham, MA, USA), 10% fetal bovine serum (FBS), Penicillin/Streptomycin(Gemini, West Sacramento, CA, USA) for 2h and then were transferred to low arginine media containing 0.2%FBS, 0.1% bovine serum albumin (BSA),and 5.5mM D-glucose (normal glucose, NG) or25mM D -glucose (high glucose, HG) for 12, 24 and 48h. For studies of the effects of arginase 1 upregulation on endothelial dependent dysfunction, isolated aortas were ligated with a 4.0 surgical line, and then adenoviral vector carrying wildtype arginase 1 (ad-Arg1-RFP,CMV-Arg1-3Flag-IRES-RFP-SV40ployA, 10^{11}pfu/mL, gift from Dr. David Fulton's lab in Augusta University) (Chandra et al. 2019; Scotland et al. 2002) was injected with M-199 medium containing adenovirus (the dose of 5μl adenovirus/cm of aorta) into the lumen of the aorta. The virus-filled vessel was incubated in NG or HG culture buffer at 37°C for 24 in hours (Scotland et al. 2002; Shosha et al. 2018). Red fluorescence protein (RFP, CMV--IRES-RFP-SV40ployA 10^{11}pfu/mL) adenoviral vector was used as control. The vessels were incubated with both ad-Arg1 and HG at the same time and all transduced vessels were then used for vascular function study. Efficiency of gene transfer was assessed using visualizing RFP fluorescence in the vessel wall, or by western blotting.

NO Production

Vascular NO production was determined by using the fluorescent NO indicator 4,5-diaminofluorescein dilacerate (No:D225, DAF-2 DA, Sigma, Saint Louis, USA)(Yao et al. 2013). Briefly, serial cross-section rings (10 mm) from frozen aorta were incubated at 37°C in HEPES buffer containing DAF-2DA (0.1μmol/L, 15 min) in the dark. To evaluate the green fluorescence is specifically produced from NO, aortic sections with DAF-2 DAwere co-incubated with L-Name (10^{-6} M, the inhibitor of nitric oxide synthesis). Sections fixed in 4% paraformaldehyde were examined under a microscope at ×100 magnification. Fluorescence intensity was qualified by the MetaMorph image analysis software (version 6.3r7). The analysis of Daf-2A fluorescence intensity was corrected for fluorescence in the presence of L-Name. For the measurement of NO production in plasma, blood plasma is separated from the fresh blood by spinning a tube of blood containing an anticoagulant at 2200-2500 rpm for 15 minutes. Because NO molecules are unstable in plasma, plasma NO production was evaluated by the content of nitrate and nitrite, which was measured by Total Nitric Oxide Assay Kit (No: S0024, Beyotime Institute of Biotechnology, Beijing, China) following the kit instruction of manufacturer (Hao et al. 2017). Briefly, the standard NaNO2 in different concentrations (0, 1, 2, 5, 10, 20, 60, 100 μM) were added into a 96-well plate along with samples (50 μL/well). Then pre-warmed Griess Reagent I and II were added into the each well (50 μL of each reagent/well). After mixed thoroughly, the absorbance of each sample was determined at the wavelength of 540 nm.

Arginase Activity Assay

arginase activity was assayed in aortic lysates in ice-cold lysis buffer (5 × 10^{-2} M Tris-HCl, 10^{-4} M EDTA and EGTA, pH 7.5) containing protease inhibitors and phosphatase inhibitors and homogenized on ice were as described (Bhatta et al. 2017). Briefly, arginase activity was evaluated by the level of urea produced from L-arginine. The supernatant (25μL) from the pulverized aorta aliquots were heated with 25μL of Tris-HCl (5 × 10^{-2} M, pH 7.5) containing MnCl2 (10mM) for 10 min at 57°C to activate arginase. 50 μL L-arginine (0.5 M in Tris-HCl) was added to the mixture and incubated at 37°C for 1 h. The hydrolysis reaction was stopped with acid solution mixture (H2SO4:H3PO4:H2O) and the mixture was heated at 100°C with a-isonitrosopropiophenone (9%, a-ISPF in EtOH, Sigma, No: 13502) for 45mins. Samples were kept in dark at room temperature for 10 mins and absorbance was measured at 540 nm. The endothelial arginase activity was calculated by the subtraction of values for the endothelium denuded aorta from values of vascular tissues with intact endothelial layer.

Quantitative reverse transcriptionPCR (Q-PCR)

Total RNA in lysates from aortic homogenates was isolated using TRIzol reagents (Invitrogen, Carlsbad, CA, USA). cDNA was generated from total RNA which was reverse transcribed with M-MLV reverse transcriptase (Invitrogen). Gene expression was determined by quantitative PCR with TaqMan @ Gene Expression

Assays(Applied Biosystems, Foster City, CA, USA)especially for arginase 1 (Hs00163660_m1,4331182) and arginase 2 (Hs00982833_m1, 4331182). All samples were performed on a StepOne Plus thermocycler (Applied Biosystems). Cycle threshold was determined for each sample, which is considered as the initial increase in fluorescence above background. GAPDH (Hs02786624_g1,4331182,TaqMan Gene@ Expression Assays) was used as internal control for normalization.

Western Blot Analysis

Protein from aortic homogenates was separated by electrophoresis on a 10% SDS–polyacrylamide pre-cast gel. Proteins were electro-blotted onto PVDF membranes (No: IPVH00010, Millipore, Shanghai, China). The blots were blocked with 5% BSA (BSA Fraction V; OmniPur) in TBST (0.2% Tween 20 in 1xTris-buffered saline). Membranes were incubated with primary antibody anti-arginase1 (1:10,000, kind gift of Dr. RW Caldwell, Augusta University) and anti β-actin (1:4000, sigma, Saint Louis, USA) prepared in 5% BSA solution overnight at 4°C. Membranes were washed (3×TBST), and incubated in secondary antibodies conjugated with horseradish peroxidase for 1 h at room temperature. Signals were detected using chemiluminescence and analyzed using densitometry.

Statistical Analysis

Experimental values of relaxation were calculated relative to the maximal changes from the contraction produced by PE taken as 100% in each tissue. Data are shown as the mean ± SEM. Concentration-response curves were fitted using a nonlinear interactive fitting program (Graph Pad Prism 7.0; GraphPad Software Inc., San Diego, CA, USA), and the maximal effect generated by the agonist (E_{max}) and the concentration of agonist that produces 50% of the maximum response (EC_{50}), which is converted to pEC50 (the negative logarithm of EC50) in bar graphs. Statistical differences were determined using analysis of variance (ANOVA) and Tukey post-test. P values < 0.05 were taken as significant. Data values for each mouse were calculated from 2 to 3 sections in image analysis studies.

References:

1 An X, Zhang M, Zhou S, Lu T, Chen Y, Yao L (2018) Xiao-Shen-Formula, a Traditional Chinese Medicine, Improves Glomerular Hyper-Filtration in Diabetic Nephropathy via Inhibiting Arginase Activation and Heparanase Expression. Frontiers in physiology 9: 1195 Doi 10.3389/fphys.2018.01195.

2 Bhatta A, Yao L, Toque HA, Shatanawi A, Xu Z, Caldwell RB, Caldwell RW (2015) Angiotensin II-induced arterial thickening, fibrosis and stiffening involves elevated arginase function. PloS one 10: e0121727 Doi 10.1371/journal.pone.0121727.

3 Bhatta A, Yao L, Xu Z, Toque HA, Chen J, Atawia RT, Fouda AY, Bagi Z, Lucas R, Caldwell RBet al (2017) Obesity-induced vascular dysfunction and arterial stiffening requires endothelial cell arginase 1. Cardiovasc Res 113: 1664-1676 Doi 10.1093/cvr/cvx164.

4 Chandra S, Fulton DJR, Caldwell RB, Caldwell RW, Toque HA (2019) Hyperglycemia-impaired aortic vasorelaxation mediated through arginase elevation: Role of stress kinase pathways. European

journal of pharmacology 844: 26-37 Doi 10.1016/j.ejphar.2018.11.027.
5 Esfandiarei M, Hoxha B, Talley NA, Anderson MR, Alkhouli MF, Squire MA, Eckman DM, Babu JR, Lopaschuk GD, Broderick TL (2019) Beneficial effects of resveratrol and exercise training on cardiac and aortic function and structure in the 3xTg mouse model of Alzheimer's disease. Drug design, development and therapy 13: 1197-1211 Doi 10.2147/dddt.S196119.
6 Faita F, Di Lascio N, Rossi C, Kusmic C, Solini A (2018) Ultrasonographic Characterization of the db/db Mouse: An Animal Model of Metabolic Abnormalities. Journal of diabetes research 2018: 4561309 Doi 10.1155/2018/4561309.
7 Hao HF, Liu LM, Pan CS, Wang CS, Gao YS, Fan JY, Han JY (2017) Rhynchophylline Ameliorates Endothelial Dysfunction via Src-PI3K/Akt-eNOS Cascade in the Cultured Intrarenal Arteries of Spontaneous Hypertensive Rats. Frontiers in physiology 8: 928 Doi 10.3389/fphys.2017.00928.
8 Kenwright DA, Thomson AJ, Hadoke PW, Anderson T, Moran CM, Gray GA, Hoskins PR (2015) A Protocol for Improved Measurement of Arterial Flow Rate in Preclinical Ultrasound. Ultrasound international open 1: E46-52 Doi 10.1055/s-0035-1564268.
9 Scotland RS, Morales-Ruiz M, Chen Y, Yu J, Rudic RD, Fulton D, Gratton JP, Sessa WC (2002) Functional reconstitution of endothelial nitric oxide synthase reveals the importance of serine 1179 in endothelium-dependent vasomotion. Circulation research 90: 904-910 Doi 10.1161/01.res.0000016506.04193.96.
10 Shosha E, Xu Z, Narayanan SP, Lemtalsi T, Fouda AY, Rojas M, Xing J, Fulton D, Caldwell RW, Caldwell RB (2018) Mechanisms of Diabetes-Induced Endothelial Cell Senescence: Role of Arginase 1. International journal of molecular sciences 19: Doi 10.3390/ijms19041215.
11 Yao L, Chandra S, Toque HA, Bhatta A, Rojas M, Caldwell RB, Caldwell RW (2013) Prevention of diabetes-induced arginase activation and vascular dysfunction by Rho kinase (ROCK) knockout. Cardiovasc Res 97: 509-519 Doi 10.1093/cvr/cvs371.
