# Supplementary figures and images for: HuangqiGuizhiWuwu Decoction Prevents Vascular Dysfunction in Diabetes via Inhibition of Endothelial Arginase 1
Source: Front Physiol. 2020 Mar 25;11:201. doi: 10.3389/fphys.2020.00201 (PMC7109290; doi:10.3389/fphys.2020.00201)

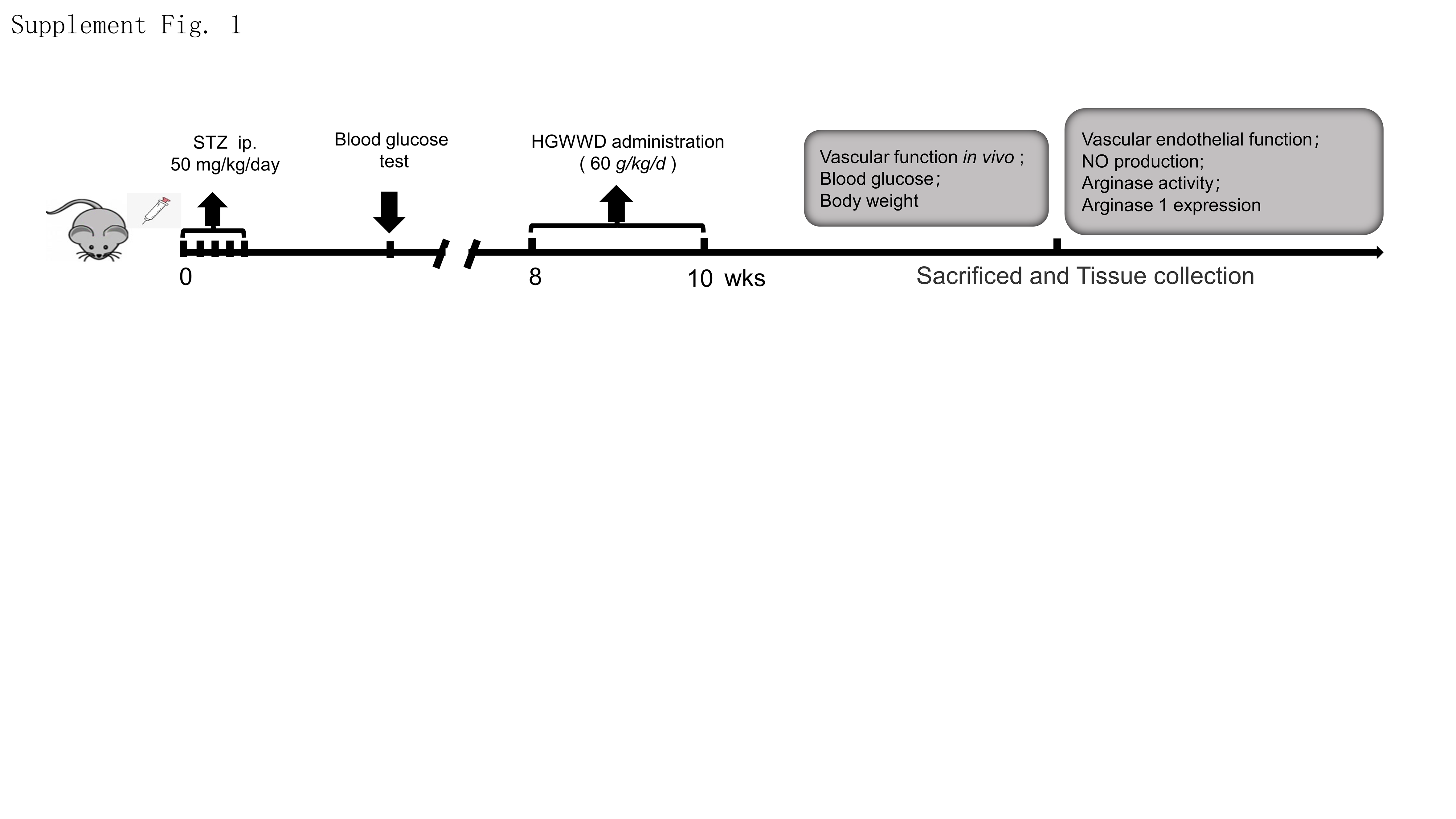

Supplement: FIGURE S1 — The experimental schedule of STZ model with HGWWD treatment. STZ, streptozotocin; HGWWD, HuangqiGuizhiWuwu Decoction; NO, nitric oxide. [file Image_1.TIF]

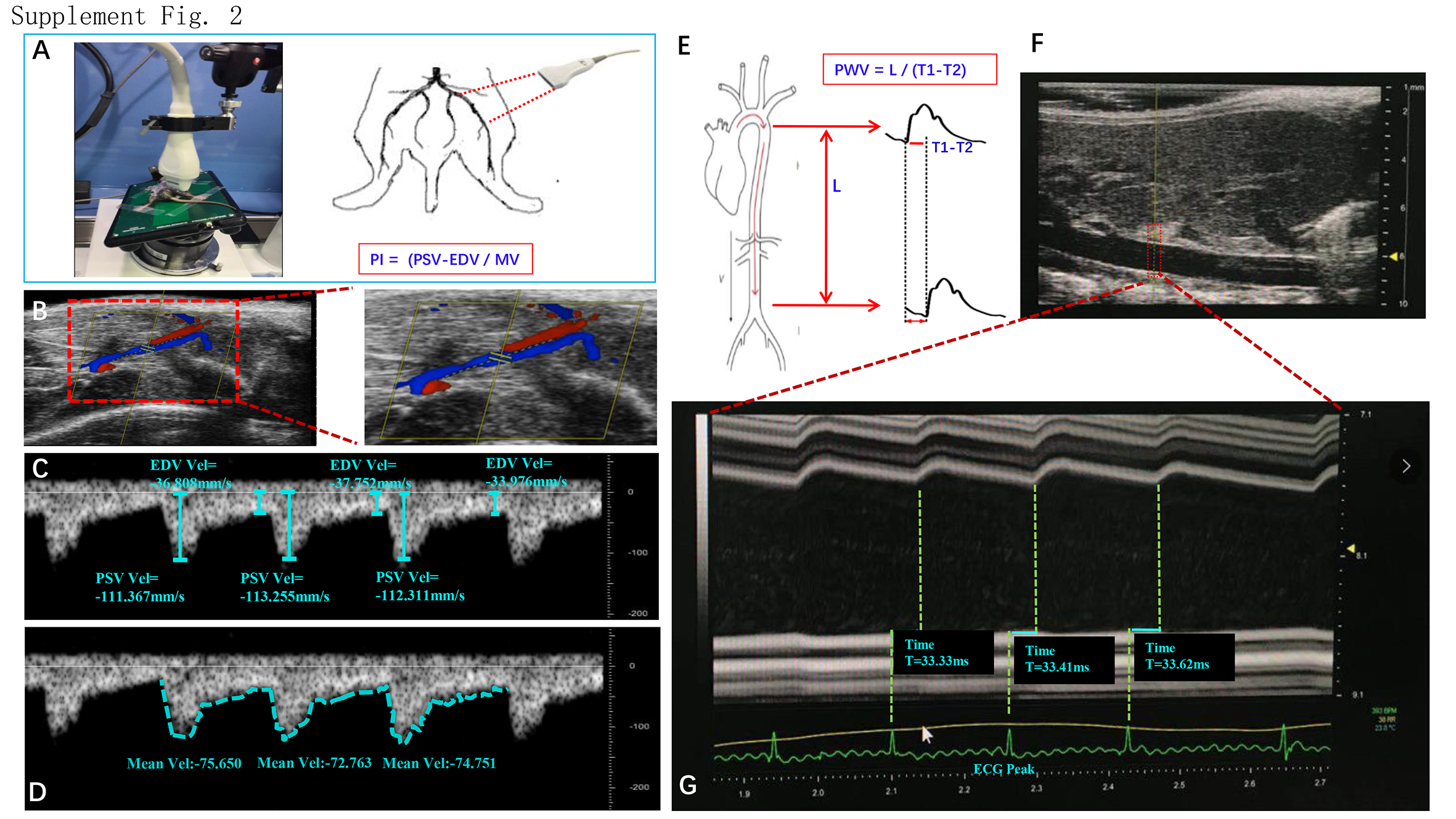

Supplement: FIGURE S2 — Schematic diagram of the measurement of mouse left femoral artery and aortic by ultrasound system. (A) Schematic diagram of mouse left femoral artery under the digital ultrasound platform. (B) The schematic diagram of mouse left femoral artery under the PW-mode combining with color flow Doppler imaging. (C) Schematic diagram of the analysis of peak systolic velocity (PSV)and end diastolic velocity (EDV) of mouse left femoral artery. (D) Schematic diagram of the analysis of mean velocity (MV) of mouse left femoral artery. (E) The schematic diagram of the measurement of pulse wave velocity (PWV). (F) The image of aorta under the M-mode. (G) Schematic diagram of the analysis of PWV under the enlarged image aortic vertical section from the part labeled by yellow dotted line. [file Image_2.TIF]

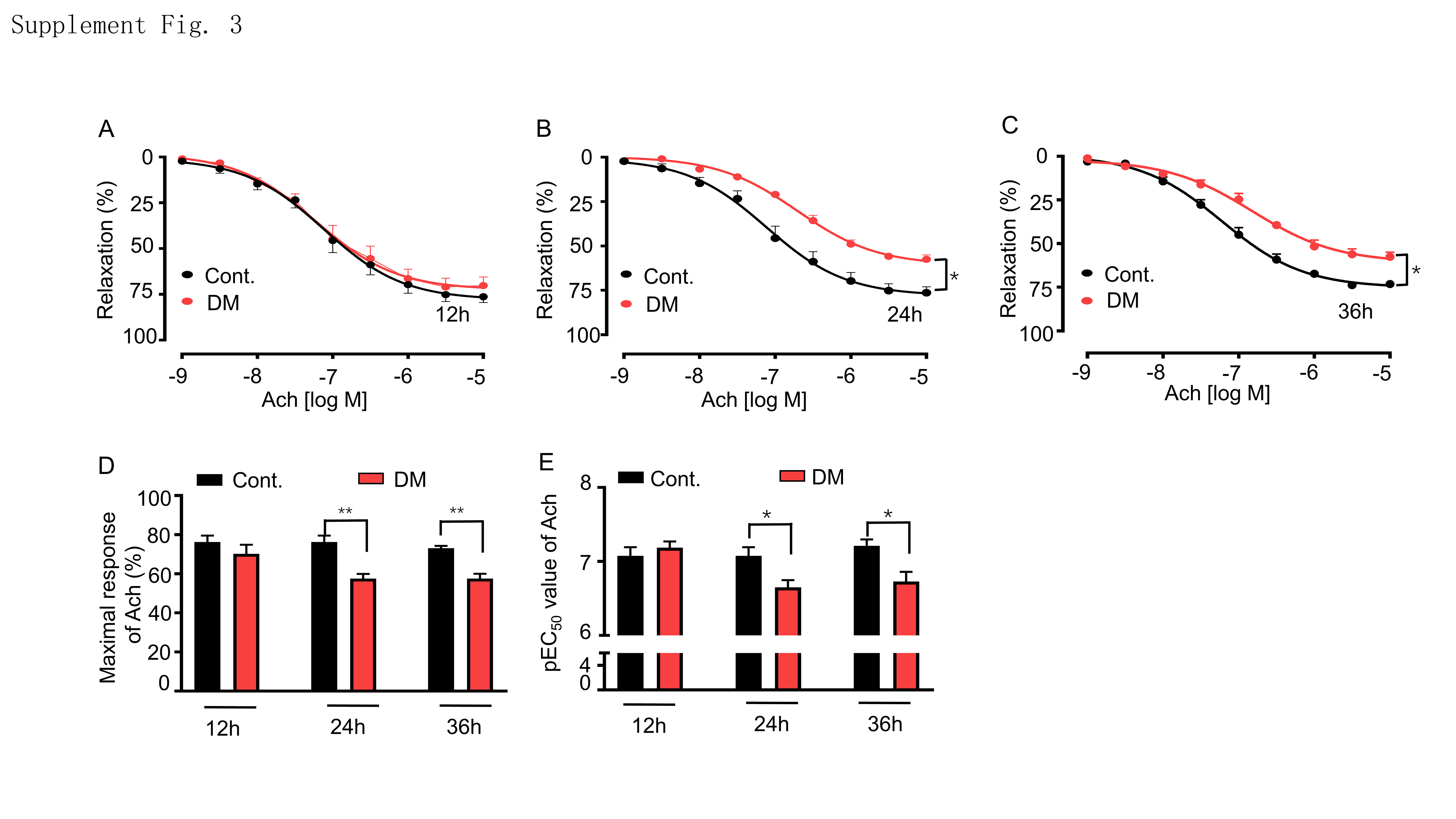

Supplement: FIGURE S3 — (A–C) Aortic endothelium-dependent relaxation curve, (D) maximal relaxation and (E) pEC50 of Ach in HG incubation for 12, 24, and 36 h. Values are presented as mean ± SEM, ∗P < 0.05 and ∗∗P < 0.001, n = 10 samples from 5 mice/group. [file Image_3.TIF]
